# A novel anatomy-based five-settlement method for endoscopic thyroid lobectomy and ipsilateral central compartment neck dissection *via* gasless unilateral axillary approach: a preliminary report

**DOI:** 10.3389/fendo.2023.1147313

**Published:** 2023-04-18

**Authors:** Jun-Na Ge, Shi-Tong Yu, Bai-Hui Sun, Zhi-Gang Wei, Zhi-Cheng Zhang, Wei-Sheng Chen, Ting-Ting Li, Shang-Tong Lei

**Affiliations:** Department of General Surgery & Guangdong Provincial Key Laboratory of Precision Medicine for Gastrointestinal Tumor, Nanfang Hospital, The First School of Clinical Medicine, Southern Medical University, Guangzhou, Guangdong, China

**Keywords:** endoscopic thyroidectomy, thyroid cancer, membrane anatomy, mesothyroid excision, surgical technique

## Abstract

**Background:**

Endoscopic thyroidectomy (ET) *via* gasless unilateral axillary (GUA) approach has been widely implemented worldwide. Based on our concept of mesothyroid excision in open surgery, we proposed a novel anatomy-based five-settlement method in ET *via* the GUA approach. This preliminary report aimed to explore the efficacy and safety of this method in patients with papillary thyroid carcinoma (PTC).

**Methods:**

PTC patients who underwent endoscopic ET and unilateral central compartment neck dissection (CCND) *via* GUA approach with the five-settlement method at the Department of General Surgery, Nanfang Hospital, Southern Medical University from March 2020 to December 2021 were retrospectively collected. The data included general clinicopathological characteristics, surgical information (including duration, complication, and clinicopathological features), and hospital stay information, and other medical records were documented.

**Results:**

In total, 521 patients underwent lobectomy and CCND under the GUA approach with the five-settlement method. The mean number of lymph nodes yielded (LNY) and positive lymph nodes (PLN) was 5.7 ± 4.3 (range, 1–30) and 1.0 ± 1.8 (range, 0–12), respectively. The incidence of transient recurrent laryngeal nerve injury was 1.1%. Chyle leakage and Horner’s syndrome respectively occurred in one patient (0.2%). Five (0.9%) patients developed a hematoma. No severe complications or conversion to open surgery have occurred.

**Conclusion:**

The five-settlement method could be implemented safely and efficiently in ET+CCND *via* the GUA approach in selected PTC patients.

## 1 Introduction

Thyroid nodules are more common in young women than in men (1). Traditional open thyroidectomy requires an obvious scar in the anterior neck that may pose severe social–physiological depressions in those patients (2, 3). It has been decades since the endoscopic technique was initially introduced to thyroid surgery; since the axillary approach was initially described by Ikeda et al. in 2002 (4, 5)and modified by Chung et al. in 2006 (6, 7), endoscopic thyroidectomy (ET) technique *via* gasless unilateral axillary (GUA) approach has been accepted for thyroid patients who want to avoid neck incision (8).

ET *via* GUA approach has several merits: a lateral view that was similar to the open surgery; less discomfort when swallowing due to the strap muscles being intact; and shorter access route required compared with other remote access approaches, for instance, the breast approach (8). However, GUA approach requires a learning curve to achieve excellence for novice surgeons (9). When the established working space is narrow, the exposure of the dorsal of the thyroid gland or inferior boundary of central compartment lymph nodes (CCLN) would be difficult. Under this circumstance, it requires the surgeons drag the thyroid for better exposure using one hand. Thus single-hand manipulation would increase surgical difficulty. Therefore, we establish an anatomy-based novel five-settlement method in ET *via* GUA approach, utilizing a specially designed retractor to achieve two-hand manipulation. The core concept of the technique is to take advantage of the cervical fascia connecting the thyroid gland and the CCLN to lift them with the retractor, then expose the posterior boundary of the thyroid gland and CCLN, and gradually dissociate the tissues from the thyroid gland and CCLN with five specific sequences to settle down each anatomical landmarks: The sternocleidomastoid muscle (SCM), the carotid sheath, the esophagus, the recurrent laryngeal nerve (RLN), and the trachea are dissociated one by one, and membrane anatomy was carried out.

In this study, we reported our experience of this novel anatomy-based five-settlement method in endoscopic thyroid lobectomy and unilateral central compartment neck dissection (ET+CCND) in papillary thyroid carcinoma (PTC) patients.

## 2 Method

### 2.1 Patients

Data on included PTC patients who underwent ET+CCND *via* GUA approach with the five-settlement method at the department of general surgery, Nanfang Hospital, Southern Medical University from March 2020 to December 2021 were retrospectively collected. The data included general clinicopathological characteristics, surgical information (including duration, complication, etc.), hospital stay information, and other medical records were documented. The time point of follow-up was December 2022.

Patient inclusion criteria included the following: (1) patients diagnosed with unilateral cT1N0M0 PTC confirmed by ultrasound-guided FNA before surgery, (2) patients who underwent GUA lobectomy with unilateral CCND, (3) patients who have strong desire for cervical cosmesis, and (4) patients who refuse to receive the active surveillance. The exclusion criteria were as follows: (1) largest tumor size > 2 cm; (2) patients with lateral neck lymph nodes metastasis, distant metastasis, or a suspicious invasion to the adjacent organs such as the RLN, esophagus, and trachea; and (3) patients who were not suitable for endoscopic surgery due to past medical history of neck surgery or irradiation. Laryngoscopy was conducted in all patients to assess the function of vocal cords before and after surgery. This study was approved by the Ethics Committee of our institution (NFEC-2021-324), and informed consent was obtained from all enrolled patients. All procedures performed in this study involving human participants were in accordance with the Declaration of Helsinki (as revised in 2013).

### 2.2 Surgical procedures

The concept of the five-settlement method of ET+CCND *via* GUA approach was similar to the idea of total mesothyroid excision as we described in open surgery (10), which is following the fascia tissue in the neck, the thyroid gland and CCLN was *en bloc* removed.

A 5-cm vertical skin incision was made starting from the anterior axillary line, along with the natural wrinkles at the axilla, and the anterior surface of the pectoralis major muscle was dissected using an electric cautery under direct vision until the SCM was exposed. A 5-mm port was placed at the groove made by the upper lateral quadrant of the breast parenchyma and the anterior axillary line, 3–5 cm away from the incision, and then a 30° rigid endoscope was inserted through the axillary incision.

### 2.3 Five-settlement method (right thyroid gland as a demonstration)

#### 2.3.1 The settlement of clavicular head of SCM

**Figure 1:**
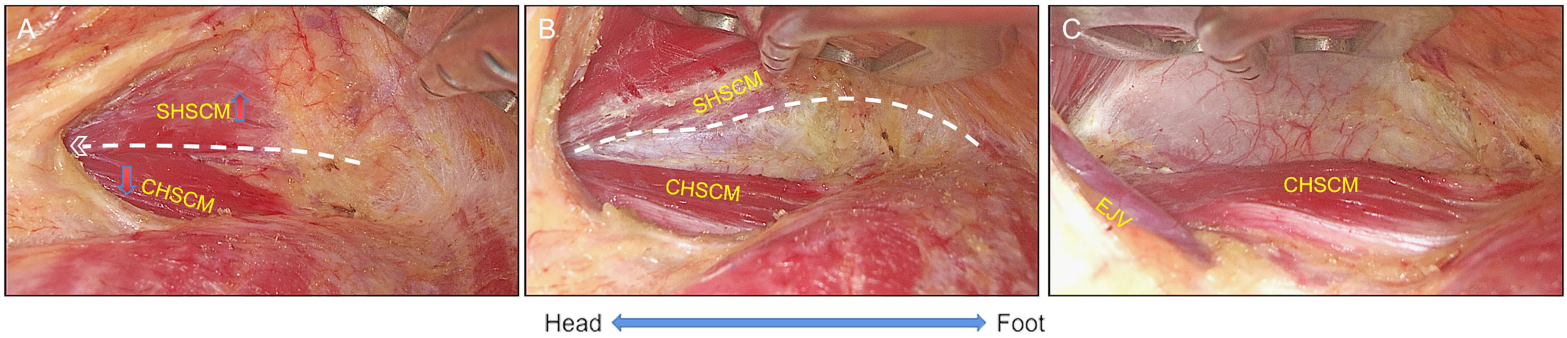
The settlement of clavicular head of SCM. **(A)** The clavicular and sternal head of sternocleidomastoid muscle (CHSCM and SHSCM) were exposed. **(B)** The space between CHSCM and SHSCM was created, and then the retractor was placed under the SHSCM (white line); **(C)** The working space was maintained by the retractor, and then deep cervical fascia was revealed and the settlement of CHSCM was done. CHSCM, clavicular head of the sternocleidomastoid muscle; SHSCM, sternal head of the sternocleidomastoid muscle; EJV, external jugular vein. White lines with angle represent cutting lines.

Remove the tissues and dissect the deep cervical fascia between the clavicular head and sternal head of SCM with harmonic scalpels (Johnson & Johnson, Cincinnati, OH, USA). By adjusting the retractor to elevate the sternal head of SCM, the clavicular head of SCM was settled (**Figure 1**).

#### 2.3.2 The settlement of carotid sheath

**Figure 2:**
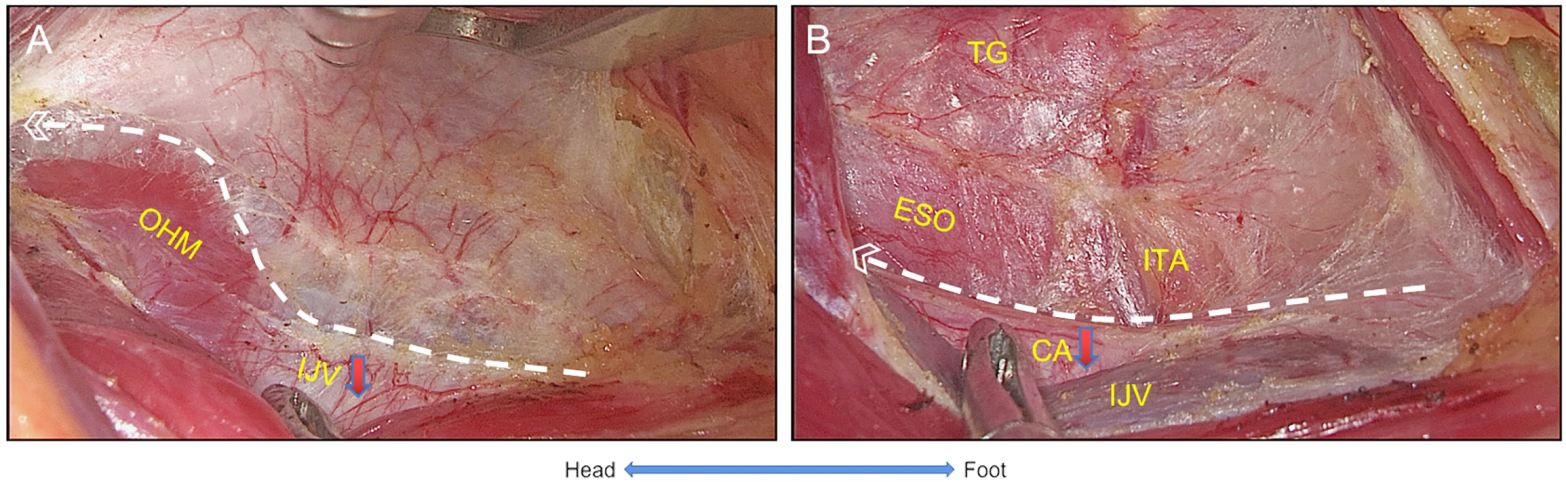
The settlement of carotid sheath. **(A)** The visceral fascia was dissected longitudinally to expose the OHM and IJV, and IJV was settled. **(B)** The inner side of the carotid sheath and the visceral fascia were dissected longitudinally, the CA was settled, and then ITA and esophagus were exposed. OHM, omohyoid muscle; IJV, internal jugular vein; CA, carotid artery; ITA, inferior thyroid artery; ESO, esophagus; TG, thyroid gland.

The harmonic scalpels were used to dissect the deep cervical fascia between the internal jugular vein (IJV) and the SCM longitudinally with grasping forceps to create tension between the CH and SCM while separating the omohyoid muscle (OHM) from the deep cervical fascia. Next, the sternothyroid muscle (STM) was grasped laterosuperiorly with the forceps and dissected laterally along the IJV, where the middle thyroid veins were identified and ligated. After that, the retractor was placed between the thyroid gland and the SCM to maintain the working space. It further expanded the retrovisceral space from the sternal notch toward the superior pole of the thyroid, while the inferior thyroid artery (ITA) and esophagus were identified and preserved (**Figure 2**).

#### 2.3.3 The settlement of esophagus

**Figure 3:**
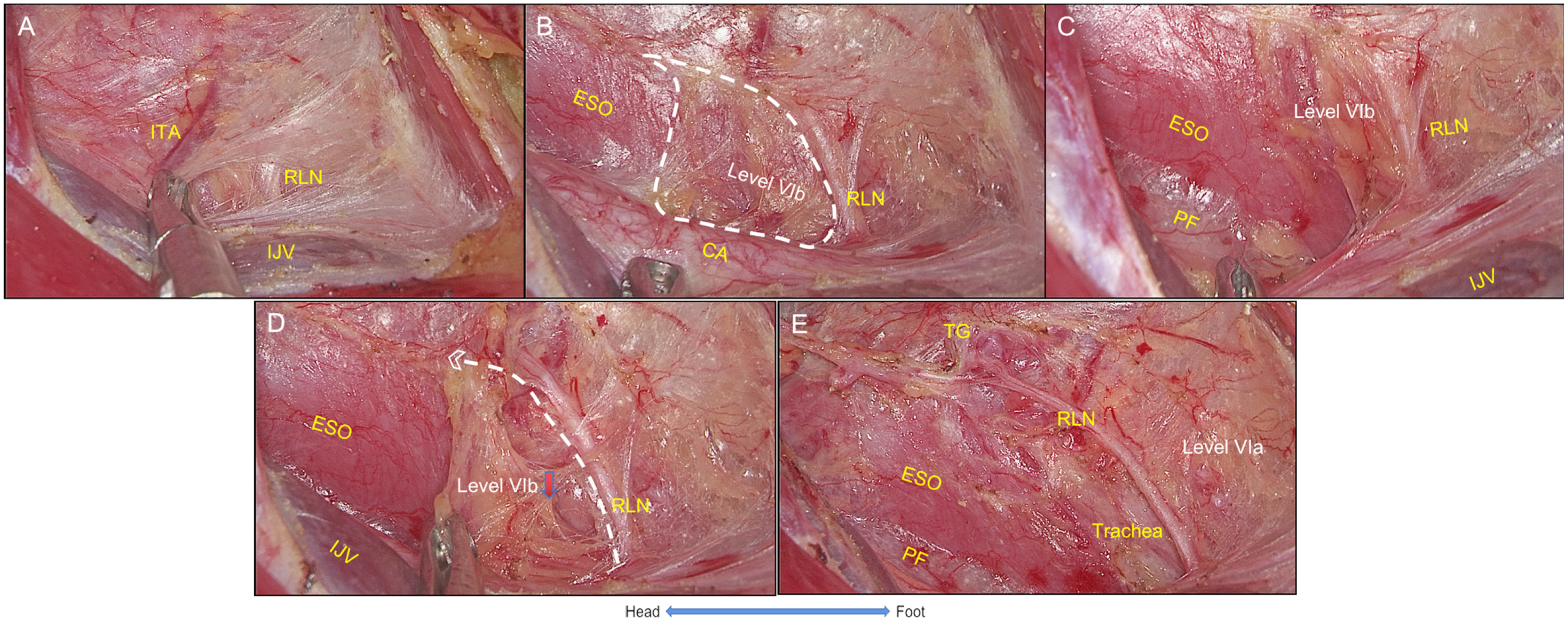
The settlement of esophagus. **(A)** The right RLN was identified at the starting point. **(B)** The buccopharyngeal fascia was dissected to expose the esophagus and level VIb, and ITA was ligated. **(C)** The posterior boundary of the surgical space should be prevertebral fascia. **(D)** The right RLN was traced toward to the ending point, while Level VIb was settled. **(E)** The settlement of esophagus was completed. RLN, recurrent laryngeal nerve; IJV, internal jugular vein; ITA, inferior thyroid artery; CA, carotid artery; PF, prevertebral fascia; ESO, esophagus; TG, thyroid gland. White lines with angle represent cutting lines.

The buccopharyngeal fascia (posterior part of visceral fascia) was dissected from clavicular level to the level of the superior pole of the thyroid gland, and the tissue above the esophagus (harboring the lymph nodes of Level VIb on the right side) was separated from the inferior to superior direction. The posterior boundary of manipulation is the prevertebral fascia. Then, RLN was exposed and located by intraoperative neuromonitoring (IONM). The NIM-Response^®^ 3.0 system was used for neuromonitoring (Medtronic Xomed Inc., Jacksonville, FL, USA) (**Figure 3**).

#### 2.3.4 The settlement of RLN

**Figure 4:**
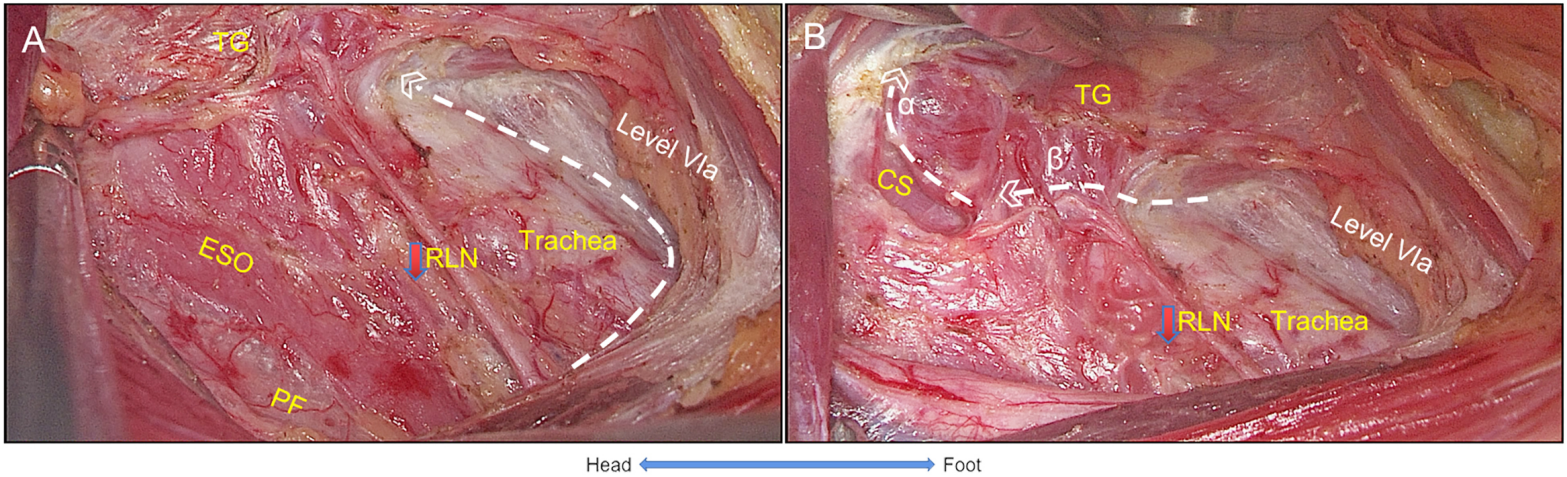
The settlement of RLN. **(A)** While the RLN was traced, the pretracheal space was expanded to reveal level VIa. **(B)** Line α shows that the superior vessels of the thyroid gland were ligated by exposing the cricothyroid space while the superior parathyroid gland was preserved, and Line β shows that the thyroid gland was resected with the entering point of RLN exposed and the RLN freed from the thyroid gland. Thus, the settlement of RLN was completed. TG, thyroid gland; RLN, recurrent laryngeal nerve; ESO, esophagus; PF, prevertebral fascia; CS, cricothyroid space. White lines with angle represent cutting lines.

Similar to open surgery, the RLN was exposed from the starting point (level of the clavicular) to the ending point (entering the larynx), and the branches innervating the trachea and esophagus should be dissected. The CCLN above the RLN was dissected along the trace of RLN by dissecting the pretrachea space. On the left side, the RLN travels longitudinally in the tracheoesophageal groove, and several holes could be made above the RLN by forceps for dissection. On the right side, after the settlement of the esophagus, the retractor was adjusted to lift the thyroid gland and CCLN upwardly, expanding the surgical plate to the level of pretrachea space. During the dissection, the ITA was ligated while the branches into parathyroid glands (PTGs) were preserved. Then, the superior thyroid vessels were individually ligated by the harmonic scalpel with the exposure of cricothyroid space, while the external branch of the superior laryngeal nerve and superior PTGs were properly preserved. Furthermore, the RLN ending point was exposed (**Figure 4**).

#### 2.3.5 The settlement of trachea

**Figure 5:**
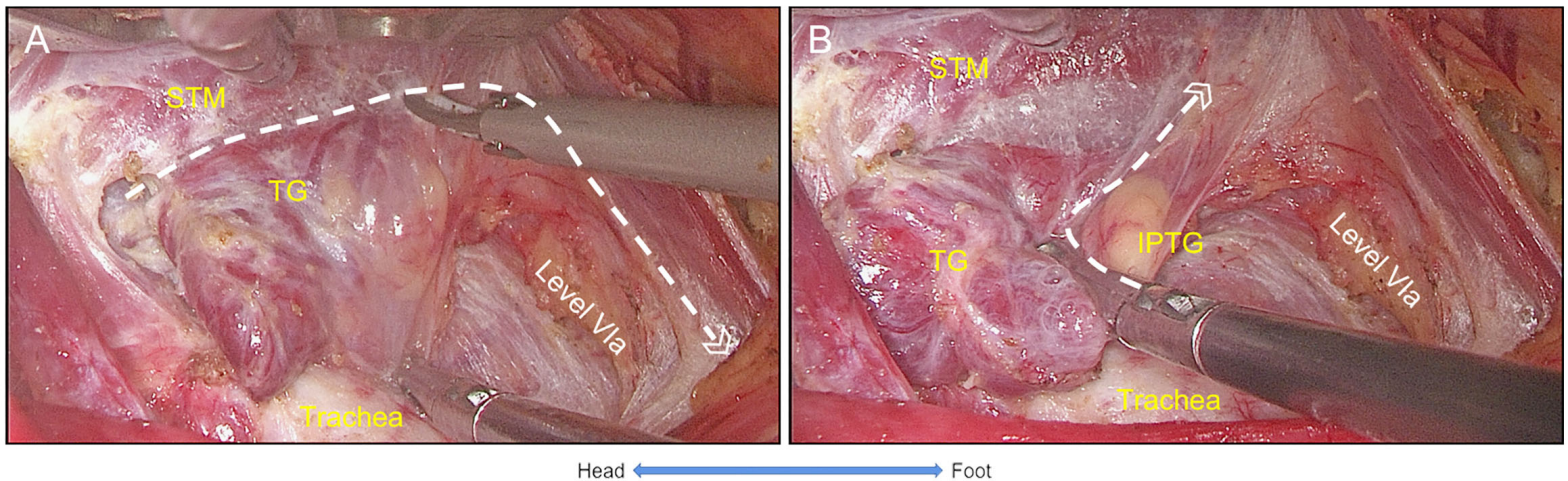
The settlement of trachea. **(A)** The thyroid gland was freed from sternothyroid muscle and trachea respectively. **(B)** Inferior parathyroid gland was identified and preserved with its vessel branches from the thymus. Thus, the settlement of trachea was completed with the *en bloc* resection of the thyroid gland and Level VIa. STM, sternothyroid muscle; TG, thyroid gland; IPTG, inferior parathyroid gland.

**Figure 6:**
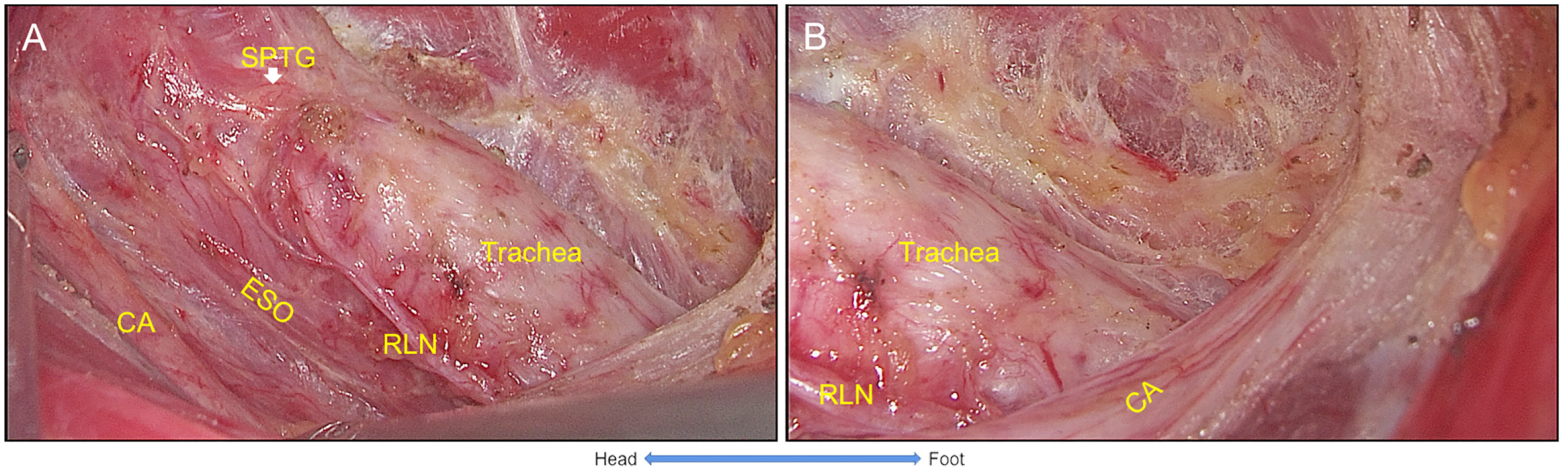
The view of unilateral ET+CCND *via* GUA approach with the five-settlement method. **(A)** The endoscopic lobectomy and central neck compartment dissection was completed with the superior parathyroid gland preserved. **(B)** The lower boundary of the central compartment was exposed (carotid artery), and the thymus and inferior parathyroid gland were preserved (IPTG within the thymus and retracted underneath the carotid artery). ET+CCND, endoscopic thyroid lobectomy, and ipsilateral central compartment neck dissection; GUA, gasless unilateral axillary; SPTG, superior parathyroid gland.

After the settlement of the RLN, the pretracheal space was entered and expanded by dissecting tissues above the RLN at the level of the clavicular. Then, prelaryngeal lymph nodes were dissected. Next, the thymus was exposed when dissecting the inferior margin of the CCLN, while inferior PTGs were preserved. Next, the STM was exposed by adjusting the retractor, and the thyroid gland and the CCLN were freed from the muscle and trachea (**Figure 5**). Last, the procedure was completed with *en bloc* removal of the unilateral thyroid gland and CCLN. The specimen was placed into a bag and retrieved from the incision (**Figure 6**).

The procedure on the left side is similar to the left side, since there is no Level VIb lymph node on the left side. Thus, the RLN was directly settled down because the nerve travels along the tracheoesophageal groove.

### 2.4 Postoperative management

Transient RLN injury was defined as vocal cord paralysis that recovered within 6 months or defined as a permanent complication. The hospital discharge was arranged according to the patients’ condition. Follow-up was usually performed at 1, 3, and 6 months and then every 6 months thereafter.

### 2.5 Statistical analysis

Continuous variables are presented as the mean ± SD or mean (range). Data were analyzed with SPSS 26.0 software (IBM Corp., Armonk, NY, USA).

## 3 Results

### 3.1 Patients’ characteristics

Five hundred twenty-one patients underwent lobectomy and CCND under the GUA approach with the five-settlement method (**Table 1**). No cases required conversion to open surgery, and all patients achieved an excellent cervical cosmetic outcome. Of the 521 patients, 108 (20.7%) were men and 413 (79.3%) were women. The mean patient age was 37.5 ± 9.6 years (range, 18–66 years). The mean BMI was 22.5 ± 3.0 (range, 16.8–30.1). The mean operative times were 90.3 ± 22.8 (range, 43–162) min. The mean bleeding volume during the surgery was 3.4 ± 2.7 (range, 2–20) ml. The mean size of tumor was 0.7 ± 0.3 (range, 0.1–1.9) cm. The mean number of lymph nodes yielded (LNY), and positive lymph nodes (PLN) were 5.7 ± 4.3 (range, 1–30) and 1.0 ± 1.8 (range, 0–12), respectively. The mean postoperative hospital stay was 3.4 ± 0.8 days (range, 3–5 days). No patient experienced structural recurrence with a mean follow-up of 18.5 ± 6.1 (range, 12–22) months.

**Table 1:** Baseline characteristics of PTC patients underwent ET *via* GUA approach with five-settlement method.

Characteristics		*n* = 521
Sex: *n* (%)		
	Female	413 (79.3%)
	Male	108 (20.7%)
Age (mean ± SD; year)		37.5 ± 9.6
BMI (mean ± SD; kg/m^2^)		22.5 ± 3.0
Operative time (mean ± SD; min)		90.3 ± 22.8
Blood loss (mean ± SD; ml)		3.4 ± 2.7
Tumor size (mean ± SD; cm)		0.7 ± 0.3
Hashimoto’s thyroiditis: *n* (%)		132(25.3%)
Lymph node yielded (mean ± SD)		5.7 ± 4.3
Positive central lymph node (mean ± SD)		1.0 ± 1.8
Postoperative hospital stay (mean ± SD; day)		3.4 ± 0.8

### 3.2 Complications

The postoperative complications of ET *via* the GUA approach are presented in **Table 2**. The incidence of transient RLN injury was 1.1%. All patients recovered within 6 months postoperatively. No patient experienced permanent RLN injury. Chyle leakage occurred in one patient (0.2%) who recovered after symptomatic treatment. One (0.2%) patient with right PTC was observed to have Horner’s syndrome on postoperative day 1; this was alleviated after 6 months with persistent slight ptosis and myosis. Five (0.9%) patients developed a hematoma. No patient experienced postoperative bleeding or conversion to open surgery. No patient experienced injuries to the esophagus or trachea.

**Table 2:** The complication rates of PTC patients underwent ET *via* GUA approach with five-settlement method.

Complication: *n* (%)		
	Transient RLN injury	6 (1.1%)
	Permanent RLN injury	0
	Postoperative bleeding	0
	Hematoma/seroma	5 (0.9%)
	Esophageal injury	0
	Trachea injury	0
	Chylous fistula	1 (0.2%)
	Horner’s syndrome	1 (0.2%)

## 4 Discussion

Boundaries have been expanded by thyroid surgery with several remote access approaches over the past decades (8). Several approaches have been developed, including transaxillary (11), breast (12), postauricular (13), and transoral approaches (14–16). Abundant evidence has been obtained for these approaches concerning their technical feasibility, safety, patient satisfaction, and cost-effectiveness.

This preliminary study comprised specific manipulation procedures with a considerable number of patients. One advantage of the procedure is that it is safe and does not require CO_2_ gas insufflation, which reduces gas-related complications while maintaining a clear operative field (17). Better exposure to the surgical field, especially the posterior boundary of the thyroid and CCLN, facilitates *en bloc* removal thyroid gland, and CCLN is another advantage of the technique. Since traditional ET, exposure to the RLN, esophagus, and inferior boundaries of CCLN is difficult due to the limitations of instruments. It requires surgeons to drag the thyroid gland with the grasp forceps. Therefore, one-hand manipulation could increase the difficulty of the surgery and also weaken the thoroughness of surgery. In the current study, we induct our technique into five specific procedures with the settlement of each anatomical landmarks. In addition, the retractor was utilized to drag the thyroid gland and CCLN upward to substitute the grasp forceps; thus, a two-hand manipulation could be achieved with a simplified procedure. Swallowing disorder following GUA thyroidectomy is less severe than open surgery. Hyun et al. reported that a comparative study showed that open surgery allowed adhesion of the strap muscle and subplatysmal muscle flap, resulting in dysphagia (18). However, the GUA approach did not require the dissection of the strap muscle. Therefore, the GUA approach provides better functional preservation of anterior neck sensory and swallow as a promising advantage. Last but not least, the GUA approach minimizes the size and visibility of skin scars by hiding them in the axilla, which is completely covered by the patient’s arm, which provides a cosmetic advantage (19).

To compare our technique with previous GUA approach reports based on the endoscopic platform, we have reviewed the literature and summarized it in **Table 3**. The mean age of patients received ranged from 35 to 42.6 (20, 23–28). The mean BMI ranging from 23.4 to 24.5 was only reported in three pieces of literature (20, 24, 27). Our enrolled patients’ age and BMI were similar. Surgical time was reported diversely, with a mean ranging from 86.9 to 193.3 min. The mean surgical time in the current study was 90.3 ± 22.8 min, which was not worse than the previous reports. Transient and permanent RLN injury was 0–6.8% and 0–2.4%, respectively. In the current study, transient RLN injury occurred in six patients (1.1%), and no permanent injury occurred. We believed that our technique could facilitate the management of RLN since the magnified view of endoscopy and better exposure of RLN provided by the technique. No postoperative bleeding was experienced, and only five cases of hematoma/seroma were similar to previous reports. Chyle leakage and Horner’s syndrome, respectively, occurred in one patient. These were relatively rare complications. We believed that the thermal damage to the cervical sympathetic nerve could explain the occurrence of Horner’s syndrome. Min et al. recommend that a safe distance to the prevertebral fascia is necessary when using a harmonic scalpel to remove the thyroid lobe (29). Roh et al. reported four (1.4%) cases of chyle leakage in 283 PTC patients undergoing thyroidectomy and CCND. They recommended that these patients should be treated with pressure dressings and a medium-chain triglyceride diet. In the current study, only one patient experienced chyle leakage and recovered within 2 days after the surgery under a symptomatic treatment.

**Table 3:** Characteristics of patients who underwent GUAET and CCND in the literature.

Authors’ name (year)	Patients	Age (years)	BMI	Time (min)	Transient RLN injury	Permanent RLN injury	Postoperative bleeding	Hematoma/seroma	Esophageal injury	Trachea injury	Number of retrieved LNs
Kang SW (2009) (7)	581 (GUA 133 cases)	36.9 ± 9.9	NA	127.0 ± 40.0	13 (6.5%)	2 (1.8%)	0	4 (0.7%)	1 (0.2%)	3 (0.5%)	4.6 ± 3.2
Cabot JC (2012) (20)	15	42.6 ± 10.0	23.4 ± 3.0	184.9 ± 26.0	0	0	0	0	0	0	NA
Lee MC (2013) (21)	84	39.4 ± 10.3	NA	193.3 ± 52.6	5 (6.0%)	2 (2.4%)	0	5 (6.0%)	0	0	NA
Kwak HY (2014) (22)	200	38.1 ± 8.4	NA	102.0 ± 33.0	5 (2.5%)	0	0	0	0	0	4.9 ± 4.2
XU JJ (2020) (23)	88	35.3 ± 9.5	NA	93.5 ± 22.0	6 (6.8%)	0	0	1 (1.1%)	0	0	4.3 ± 3.0
Xu SY (2021) (24)	35	39.0 ± 9.0	24.0 ± 3.0	116.0 ± 14.0	2 (5.7%)	0	1 (2.9%)	0	0	0	4.0 ± 1.6
Cong R (2022) (25)	51	35.0 (SD was not given)	NA	141.6 ± 34.4	2 (3.9%)	0	0	1 (2.0%)	0	0	3.2 ± 3.2
Sun BT (2022) (26)	105	37.1 ± 8.5	NA	86.9 ± 31.3	5 (3.9%)	0	0	0	0	0	5.1 ± 2.3
Chen DQ (2022) (27)	55	41.6 ± 7.3	24.5 ± 1.3	109.0 ± 10.3	2 (3.6%)	1 (1.8%)	0	0	0	0	NA

NA, not available.

The number of lymph node yielded (LNY) was reported with a mean ranging from 3.15 to 5.13. In this cohort, the mean of LNY and PLN was 5.7 ± 4.3 and 1.0 ± 1.8, retrieved from a unilateral CCND, respectively. The optimal LNY in bilateral CCND to decrease the chance of recurrence in the central neck for PTC was 11 (30), consistent with the current study. We believe that, in the five-settlement method, the inferior thyroid artery was easier to identify, and deep cervical fascia and the cervical segment of the esophagus were then exposed. Then, the posterior boundary of the thyroid gland and CCLN was managed with a better surgical view. Therefore, membrane anatomy was carried out to dissect the thyroid and CCLN between fascial tissue with less bleeding and complications, as we initially described in open surgery and ET (3, 10).

The current study has several limitations, including the discrepancy in the cohorts and the short follow-up duration. The present cohort included patients in the early stage. Therefore, a prospective comparison study is warranted to evaluate further the effectiveness of the technique with open study or other endoscopic techniques.

In summary, the five-settlement method is an innovative and effective option for ET in PTC patients.

## Data availability statement

The raw data supporting the conclusions of this article will be made available by the authors, without undue reservation.

## Ethics statement

The studies involving human participants were reviewed and approved by Ethics Committee of Nanfang Hospital. The patients/participants provided their written informed consent to participate in this study.

## Author contributions

All authors made substantive intellectual contributions to this study to qualify as authors. S-TL conceived of the design of the study. J-NG modified the design of the study. S-TL, S-TY, J-NG, and B-HS performed the study, collected the data, and contributed to the design of the study. Z-GW and Z-CZ analyzed the data. S-TY drafted the manuscript. W-SC and T-TL edited the manuscript. All authors read and approved the final manuscript. All authors have agreed to be accountable for all aspects of the work in ensuring that questions related to the accuracy or integrity of any part of the work are appropriately investigated and resolved.
